# Targeted anti-CCP testing to identify people at risk of rheumatoid arthritis in primary care: an early economic evaluation

**DOI:** 10.1093/rap/rkag111

**Published:** 2026-09-12

**Authors:** Alison F Smith, Bethany Shinkins, Elizabeth M A Hensor, Michelle Wilson, Anna M Anderson, Rebecca Stack, Paul Emery, Kulveer Mankia, Heidi J Siddle

**Affiliations:** Leeds Institute of Health Sciences, University of Leeds, Leeds, UK; NIHR HealthTech Research Centre in Accelerated Surgical Care, Leeds, UK; Leeds Institute of Health Sciences, University of Leeds, Leeds, UK; Warwick Medical School, University of Warwick, Coventry, UK; Leeds Institute of Rheumatic and Musculoskeletal Medicine, University of Leeds, Leeds, UK; NIHR Leeds Biomedical Research Centre, Chapel Allerton Hospital, Leeds, UK; Leeds Institute of Rheumatic and Musculoskeletal Medicine, University of Leeds, Leeds, UK; NIHR Leeds Biomedical Research Centre, Chapel Allerton Hospital, Leeds, UK; Leeds Institute of Health Sciences, University of Leeds, Leeds, UK; NIHR HealthTech Research Centre in Accelerated Surgical Care, Leeds, UK; Leeds Institute of Rheumatic and Musculoskeletal Medicine, University of Leeds, Leeds, UK; Three Counties Medical School, University of Worcester, Worcester, UK; Warwick Medical School, University of Warwick, Coventry, UK; Leeds Institute of Rheumatic and Musculoskeletal Medicine, University of Leeds, Leeds, UK; Leeds Institute of Rheumatic and Musculoskeletal Medicine, University of Leeds, Leeds, UK; NIHR Leeds Biomedical Research Centre, Chapel Allerton Hospital, Leeds, UK; Leeds Institute of Rheumatic and Musculoskeletal Medicine, University of Leeds, Leeds, UK; NIHR Leeds Biomedical Research Centre, Chapel Allerton Hospital, Leeds, UK

**Keywords:** rheumatoid arthritis, anti-cyclic citrullinated peptide (anti-CCP) antibodies, prediction model, economic evaluation

## Abstract

**Objectives:**

To undertake an early economic evaluation of anti-cyclic citrullinated peptide (anti-CCP) antibody testing scenarios in primary care, to improve identification of individuals at risk of rheumatoid arthritis (RA).

**Methods:**

A discrete event simulation (DES) model was developed to track a hypothetical cohort of 10 000 patients from primary care presentation with musculoskeletal (MSK) symptoms without synovitis through to secondary care rheumatology services. Multiple anti-CCP testing scenarios were evaluated, including testing all patients on first presentation and targeted testing using a novel anti-CCP prediction model. Patients with positive anti-CCP results were referred to rheumatology services. The base-case assumed no primary care anti-CCP testing with no immediate referrals. Cost-effectiveness was assessed according to the additional cost to the healthcare system required to immediately refer one patient with imminent inflammatory arthritis (IA) (developing RA or other IA within 1 year of presentation).

**Results:**

Anti-CCP testing in all patients with MSK symptoms leads to 52.7% of those with imminent IA being immediately referred, versus 23.8% with targeted testing. Average per-patient short-term healthcare costs were £77.44 (no testing), £127.83 (testing all) and £87.54 (targeted testing). Versus no testing, additional costs of £5038 (testing all) or £2234 (targeted testing) were incurred per additional patient with imminent IA immediately referred. Targeted testing incurred more favorable cost-per-referral results than indiscriminate testing over all analyses.

**Conclusion:**

Targeted anti-CCP testing is a potentially cost-effective strategy to identify patients at risk of RA and enable prompt referral. Investigation of long-term costs and outcomes is warranted to determine the optimal testing strategy.

Key messages

- The anti-CCP test can identify patients at risk of rheumatoid arthritis in primary care, but testing all patients who present with musculoskeletal symptoms is unlikely to be feasible.
- An anti-CCP prediction model has been previously developed to identify patients likely to test positive on anti-CCP testing in primary care.
- Targeted testing with the anti-CCP prediction model is potentially cost-effective, indicating that further research on long-term outcomes is warranted.

## Introduction

Rheumatoid arthritis (RA), a type of inflammatory arthritis (IA), is a chronic, multi-system autoimmune disease characterized by painful swelling and destruction of synovial joints, which can lead to disability, co-morbidities (including cardiovascular and lung disease) and premature mortality [1, 2]. In people with RA, circulating anticitrullinated protein antibodies (ACPA) usually precede the onset of clinically apparent arthritis (synovitis) by several months or years, and trials have suggested that intervening in the pre-arthritis at-risk stage may delay or even prevent RA onset [3–5]. Recent evidence demonstrates that treatment-related costs and frequency of use are lower in RA patients who had a shorter symptom duration (<12 weeks), and that treatment with methotrexate may be cost-effective in people with clinically suspect arthralgia who are at risk of developing RA [6, 7]. Further research is required, however, to evaluate the cost-effectiveness of diagnostic interventions for the earlier identification of those at risk of RA [6].

The anti-cyclic citrullinated peptide (anti-CCP) test is available to identify patients with ACPA, however current guidance from the National Institute of Health and Care Excellence (NICE) in the UK only recommends anti-CCP testing in people who already have clinically apparent synovitis if they are negative for rheumatoid factor, or following diagnosis [8]. Anecdotal reports suggest there are wide variations in access to and use of the anti-CCP test in the UK primary care. Targeted anti-CCP testing in people who present with new non-specific musculoskeletal (MSK) symptoms in primary care (around 3% of whom are likely to test positive for anti-CCP) provides an opportunity to identify people at risk of developing RA or another IA before they develop synovitis [9–11]. These individuals may then be promptly referred to rheumatology services for monitoring, early diagnosis and rapid treatment initiation [12, 13], thus avoiding the current process of multiple primary care appointments and significant delays [14].

In previous work, we developed a prediction model (the ‘anti-CCP prediction model’) to identify patients presenting to primary care with new onset non-specific MSK symptoms without synovitis who are likely to be anti-CCP positive and therefore at risk of RA [15, 16]. The prediction model was developed using the Leeds Co-ordinated Programme to Prevent Arthritis cohort—a large (6879 participants), longitudinal cohort of individuals with new-onset non-specific MSK symptoms which included baseline anti-CCP testing. It includes 11 predictors (male sex, first degree relative with RA, ever smoked and joint pain in: back, neck, shoulders, wrists, hands/fingers, thumbs, knees, feet/toes) and for patients with an elevated score clinicians would be prompted to refer the patient for an anti-CCP test which, if positive, could support referral to secondary care [15, 16]. Previous analysis indicated that, of those over the threshold for anti-CCP testing based on this prediction model, 6.68% (30/445) would be expected to develop IA within 12 months (56.8% [25/44] in the anti-CCP positive group *vs*.s 1.2% [5/405] in the anti-CCP negative group), compared with 0.9% (18/2031) of those below the testing threshold [15, 16].

The question remains as to whether investment in the anti-CCP prediction model and associated anti-CCP testing could be considered cost-effective within the context of constrained health care budgets and referral pathways. Data to inform economic evaluation in this area is currently limited. We therefore undertook an early, exploratory economic evaluation aimed at assessing the impact of targeted anti-CCP testing using the anti-CCP prediction model on (i) NHS healthcare costs relating to the initial short-term diagnostic pathway and (ii) patients’ time to attending rheumatology services. We explore whether such a strategy could be cost-effective and identify key areas of uncertainty for consideration in future research.

## Methods

### Model structure and outcomes

A computer-based Discrete Event Simulation (DES) model was constructed using Simul8 software to mimic the journey of a hypothetical cohort of 10 000 patients with new onset, non-specific MSK symptoms without synovitis, from first primary care presentation through to attendance at secondary care rheumatology services and diagnosis [17]. This evaluation did not involve any patient recruitment or randomization; as such, no ethical review was required for this study.

The DES approach simulates individual patient pathways, with each patient’s sequence of events dictated by individually sampled waiting times and probability variables, based on data from the literature. Figure 1 presents a simplified model schematic. The model has no fixed time horizon—rather the simulation ends when all patients destined to be referred to rheumatology services have been seen by a rheumatologist. The total time it takes patients to reach rheumatology services in the model is dictated by two key delay variables representing (i) the waiting time to receive the referral and (ii) the subsequent time taken to be seen in secondary care (full details under Model Parameters).

**Figure 1:**
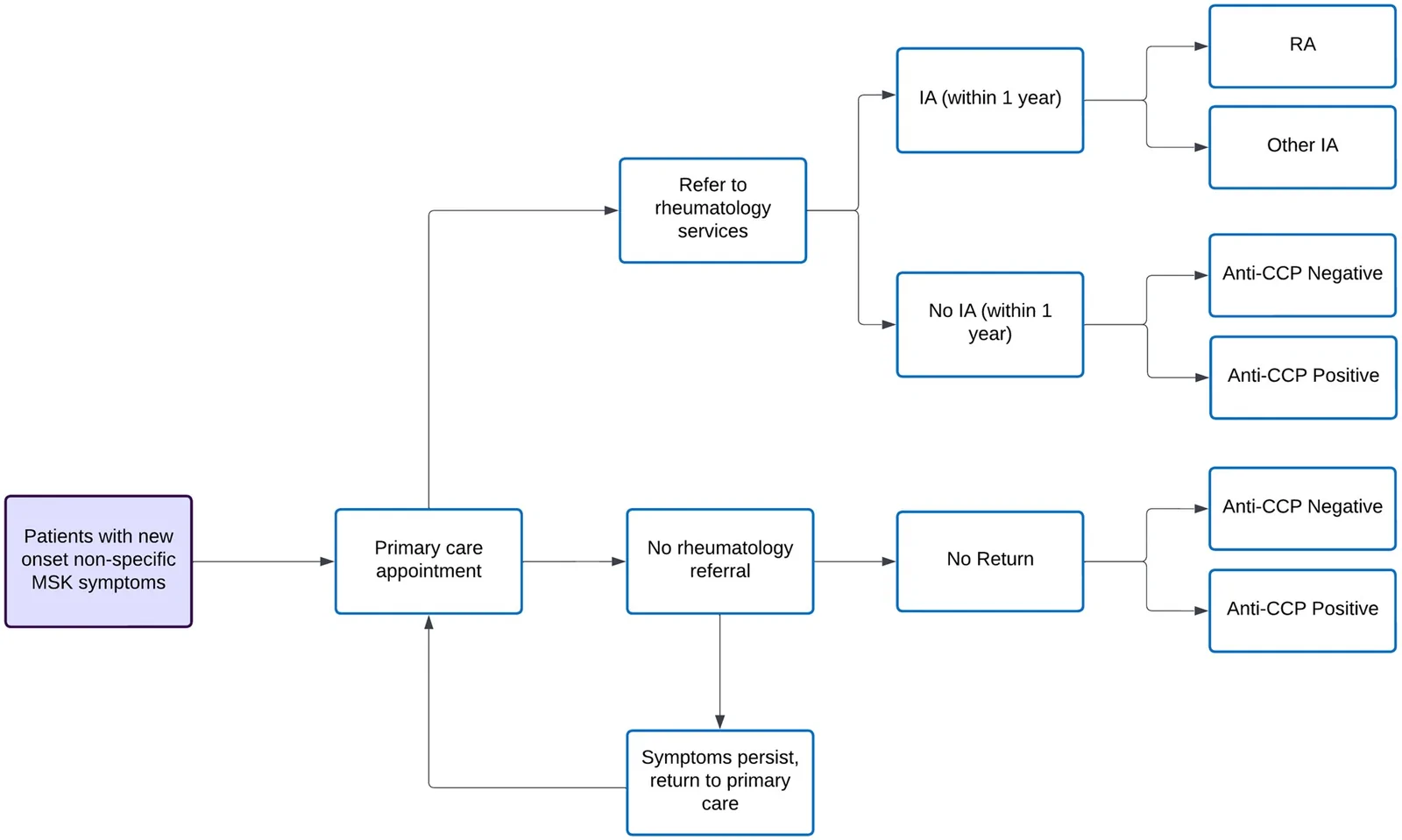
Model figure. Patients with new onset non-specific MSK symptoms arrive for their first primary care appointment. Under anti-CCP testing scenarios, patients may be immediately referred to rheumatology services upon receipt of a positive anti-CCP test. Without anti-CCP testing, no patients are referred immediately and instead patients may return for a later referral following persistent symptoms. Under the targeted testing strategy, the anti-CCP prediction model prompts targeted anti-CCP testing, with only those patients expected to be positive according to the prediction model undergoing anti-CCP testing (see Fig. 2). The model tracks patients for 1 year, at which point patients may have confirmed RA, other IA or have no confirmed IA (with positive or negative anti-CCP status). Note that patients anti-CCP status may or may not be observed, depending on the testing strategy adopted

Key modelled outcomes included: (i) the percentage of patients referred immediately (i.e. at first primary care presentation), (ii) time to attendance at rheumatology services, (iii) number of primary care appointments and (iv) direct short-term healthcare costs. We assessed the short-term cost-effectiveness of different testing scenarios by asking: ‘Compared with no testing, how much extra does the NHS need to spend to correctly refer one additional patient with imminent IA under this scenario?’ where *imminent RA* is defined as patients who develop RA or other IA within 1 year of first primary care presentation. More formally, short-term cost-effectiveness was assessed using the incremental cost-effectiveness ratio (ICER), calculated pairwise for each testing scenario as the additional cost to the healthcare service required to refer one additional patient with imminent IA, versus the model base-case arm (discussed below).

Based on variation in current clinical management, eight different anti-CCP testing scenarios were evaluated, applying different levels of testing. Each scenario was evaluated independently using the same hypothetical cohort and underlying disease incidence assumptions. Differences in outcomes therefore arose solely from the alternative testing and referral pathways modelled, rather than from differences in the underlying patient population.

For current standard of care, an observational study conducted in the UK in 2019 demonstrated that patients with RA or unclassified arthritis typically experience multiple GP appointments (median 4) and significant delay between first presentation and GP decision to refer (median delay 6.9 weeks; IQR 2.3–30.2) [14]. The model base-case arm (representing standard care) therefore assumes no anti-CCP testing would be undertaken on initial primary care presentation and no patients thus receive an immediate referral. Instead, patients with imminent IA are assumed to return for additional appointments before receiving a delayed referral (e.g. at the point at which they develop clinical synovitis). Under the primary intervention scenario of the model—the targeted testing strategy—the anti-CCP prediction model prompts targeted anti-CCP testing at the patients’ first primary care appointment, with only those patients expected to be positive according to the prediction model undergoing anti-CCP testing (see Fig. 2). All patients with a positive anti-CCP test were assumed to be referred to rheumatology services.

**Figure 2:**
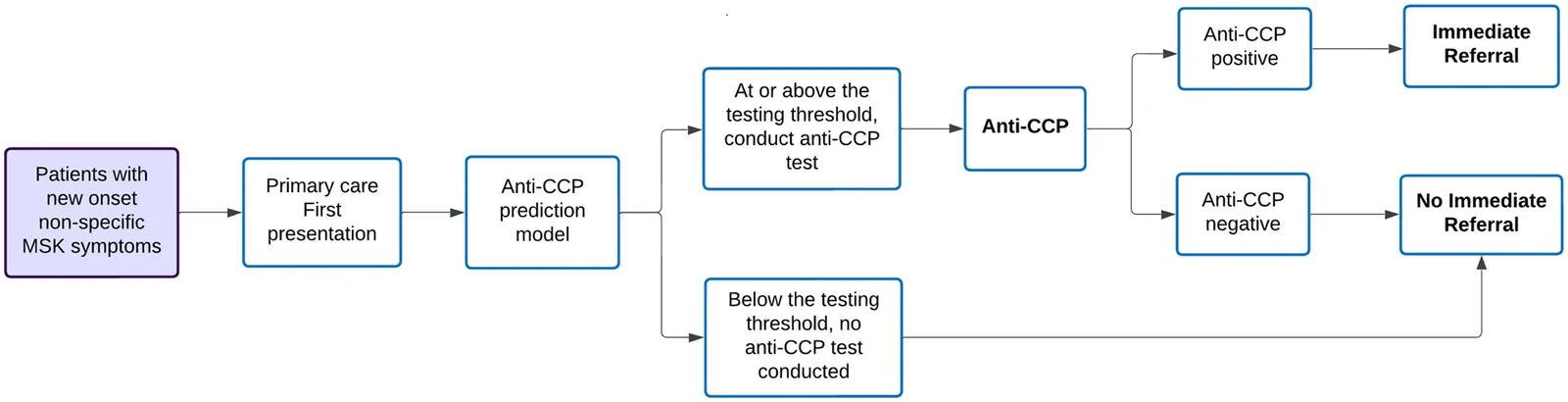
Targeted anti-CCP testing pathway—immediate referrals logic. Under the targeted testing strategy, the anti-CCP prediction model identifies which patients are likely to be anti-CCP positive, with those patients above the anti-CCP prediction model threshold undergoing anti-CCP testing. Only patients who receive a positive anti-CCP test are assumed to receive an immediate referral to rheumatology services; the remaining patients may return for a later referral if they experience persistent symptoms

Additional anti-CCP testing scenarios were considered, applying seven alternative levels of (non-targeted) anti-CCP testing upon patients’ first presentation, ranging from 10% to 100% having an anti-CCP test. In practice, primary care clinicians would decide whether or not to use the anti-CCP test based on patient characteristics (e.g. number of joints affected); however, in the absence of data to inform selective testing, the use of anti-CCP testing was assumed to be applied randomly across the modelled population.

The model assumes that all patients destined to develop IA (including RA) within 1 year of their first primary care presentation (the imminent IA population) will be referred to secondary care, either immediately following a positive anti-CCP test or following further primary care appointments for those not tested or who receive a negative anti-CCP test. There is a lack of data to inform what happens to other patients in the model (i.e. those who do not develop IA within 1 year). Most patients with non-specific MSK symptoms without synovitis will not go on to develop RA or other IA, and may receive alternative care or specialist referrals. In the base-case analysis, across all testing scenarios it was assumed that 10% (range 5–15%) of patients with negative anti-CCP status would return for a delayed rheumatology referral; investigations and referrals relating to other (non-rheumatology) specialist services (where many of these patients may end up) were not captured. For patients with positive anti-CCP status (a proportion of whom are not immediately referred, in those scenarios where universal testing is not undertaken) and who do not develop IA within 1 year of presentation, it was assumed that 20% (range 10–30%) would return for a delayed referral. These values were altered in sensitivity analyses.

Following referral, patients attend an appointment with a rheumatology specialist. Those who develop IA within 1 year are assumed to be identified in secondary care, whilst those with positive anti-CCP status will be known following testing and different monitoring scenarios may be considered for those patients (monitoring scenarios are not modelled in this analysis). Anti-CCP status in patients not referred to secondary care will only be known for those patients who were tested upon first primary care presentation and received a negative test result (anyone with an observed positive anti-CCP result is assumed to be referred).

#### Model parameters

Model parameters are reported in Table 1.

**Table 1:** Model base-case parameters.

Parameter	Mean Value	Distribution in model	Source
**IA/RA incidence**			
Incidence of IA (within 1 year)	1.90%	Beta (110, 5681)	Garcia-Montoya *et al*. (2022) [9]
% RA in patients with IA (anti-CCP positive)	92.6%	Beta (63, 5)	
% RA in patients with IA (anti-CCP negative)	39.6%	Beta (21, 32)	
**Test accuracy**			
Anti-CCP prediction model sensitivity (threshold ≥11)	38.42%	Beta (78, 125)	Siddle at al. (2024) [15]
Anti-CCP prediction model specificity (threshold ≥11)	81.88%	Beta (5466, 1210)	
Anti-CCP test sensitivity	51.82%	Beta (57, 53)	Garcia-Montoya *et al.* (2022) [9]
Anti-CCP test specificity	98.35%	Beta (5587, 94)	
**Time to event parameters (weeks)**			
Primary care referral delay (patients referred on 1^st^ appt)	5.96	LN (meanlog = 0.19185, sdlog = 1.92616)	Stack *et al.* (2019) [14]
Primary care referral delay (patients referred later)	33.47	LN (meanlog = 2.20286, sdlog = 1.75973)	Stack *et al.* (2019) [14]
Number of primary care appointments (patients referred later)	4.54	Linear regression model (see Supplementary Data S1, available at *Rheumatology Advances in Practice* Online)	Stack *et al.* (2019) [14]
Secondary care delay	6.31	LN (meanlog = 1.49710, sdlog = 0.82111)	Stack *et al.* (2019) [14]
**Primary care probabilities**			
Anti-CCP testing: % CRP and RF tests ordered with anti-CCP test	50%	Fixed	Assumptions informed by clinical opinion
No anti-CCP testing, referred patients: % CRP and RF conducted in primary care prior to referral	50%	Fixed	
% persistent RA symptoms (anti-CCP positive)	20%	Triangular (10, 20, 30)	
% persistent RA symptoms (anti-CCP negative)	10%	Triangular (5, 10, 20)	
% receiving primary care follow-up appt. after anti-CCP test	20%	Triangular (10, 20, 30)	
**Secondary care investigations**			
% receiving x-ray	35%	Triangular (25, 35, 50)	Clinical opinion
% receiving ultrasound	60.7%	Beta (54, 35)	Kulveer Mankia *et al.* (2020) [19]
% receiving MRI	2.3%	Beta (2, 86)	
**Costs (2024 prices)**			
Anti-CCP	£8.74	Fixed	NHS National Cost Collection (2023) [DAPS06] [21]
CRP	£8.74	Fixed	
RF	£8.74	Fixed	
Phlebotomy	£5.40	Fixed	As above [DAPS08] [21]
GP appointment (10 min)	£57.63	Fixed	PSSRU Manual (2023) [20]
FCP appointment (20 min)	£22.00	Fixed	
GP Review of anti-CCP result	£28.81	Triangular (11.53, 28.81, 46.10)	PSSRU Manual (2023) [20]. Distributions assume 3, 5 and 8 min of clinician time required.
FCP Review of anti-CCP result	£5.50	Triangular (2.2, 5.5, 8.8)	
Specialist referral appointment	£313.07	Fixed	NHS National Cost Collection (2023) [WF01B; DAPS05; PF; RD40Z; RD01A] [21]
Routine bloods	£3.40	Fixed	
X-ray (Hands and Feet)	£32.16	Fixed	
Ultrasound	£89.36	Fixed	
MRI	£216.04	Fixed	

Anti-CCP, anti-cyclic citrullinated peptide; CRP, C-reactive protein; FCP, first contact physician; GLM, generalized linear model; GP, general practitioner; IA, inflammatory arthritis; LN, lognormal; RA, rheumatoid arthritis; RF, rheumatoid factor; MRI, magnetic resonance imaging; meanlog, mean S.D. of the distribution on the log scale; sdlog, S.D. of the distribution on the log scale.

##### Disease incidence and test accuracy

Incidence of IA was based on a UK prospective observational study which assessed the association between baseline factors and risk of progression to IA (including RA) for patients with new onset, non-specific MSK symptoms (excluding clinical synovitis) recruited from 312 primary care centers (2007–2019) [9]. Of 5791 patients, 110 (1.90%) developed IA within 1 year. The probability of RA in the model was assigned according to patients’ anti-CCP status, based on the fact that 93% (63/68) of the study participants who developed IA (at any time point) and had positive anti-CCP status met the ACR/EULAR 2010 criteria for RA, compared with 40% (21/53) in those who were anti-CCP negative [9].

The same study informed the accuracy of the anti-CCP test. Of 110 patients who developed IA within 1 year, 57 had positive baseline anti-CCP (51.82% sensitivity); whilst of 5681 patients who did not develop IA within 1 year, 5587 had negative anti-CCP status (98.35% specificity) [9].

The accuracy of the anti-CCP prediction model for predicting anti-CCP positivity was taken from the Leeds CCP Cohort study—a prospective, observational primary care cohort study which recruited individuals through the UK Primary Care Clinical Research Network (2007–2020) [15]. Based on 6879 included participants, the anti-CCP prediction model identified 78/203 patients with positive anti-CCP (38.42% sensitivity) and 5466/6676 patients with negative anti-CCP (81.88% specificity), using a threshold of 4% (corresponding to an anti-CCP prediction model score ≥11). An alternative threshold of 3% (prediction model score ≥8) achieved 60.10% (122/203) sensitivity and 60.22% specificity (4020/6676), and was considered in sensitivity analysis [15].

##### Time to referral and secondary care

A patient’s journey from primary to secondary care is composed of two key delay (waiting time) variables: *primary care referral delay*—the time from a patient’s first primary care appointment to the time the primary care clinician decides to refer that patient to secondary care; and *secondary care delay*—the time between referral and the patient being assessed by rheumatology services.

A study by Stack *et al.* [14] in 2019 reported delay data on 822 newly presenting adults with either RA or unclassified arthritis, based on patient-reported questionnaires. Patients were recruited consecutively from secondary care rheumatology departments following a primary care referral, across 34 NHS Trusts in England and Scotland (2011–14). Further information was sought from the study authors, and a modified dataset including data for 820 patients was received. For each time-to-event variable, statistical distributions (exponential, Weibull, gamma and lognormal) were applied using the *fitdistrplus* package in R [18]. Based on standard statistical measures (AIC and BIC) the best fitting distribution in each case was the lognormal distribution, which was used in the model to sample patients’ delay variables. A sensitivity analysis was conducted using the Weibull distribution (the next best alternative). Figures showing the theoretical densities of the alternative parameterizations against the observed data are provided in Supplementary Figs S1–S3, available at *Rheumatology Advances in Practice* Online.

Primary care referral delay was calculated separately for patients referred following a single primary care visit versus those referred after multiple visits. Testing scenarios in the model could impact patients’ time to secondary care by altering the probability of immediate referral (associated with significantly shorter primary care referral delay versus later referrals).

##### Investigations and appointments

Primary care anti-CCP testing was assumed to require one blood test appointment and an additional 5 min (range 3–8) of clinician review time. Based on discussions with clinicians and patients from the study advisory group, 20% of patients were conservatively assumed to attend a primary care follow-up appointment (face-to-face) after an anti-CCP test (regardless of the test result). Follow-up appointments were assumed not to impact patients’ time to secondary care (for those referred) but incurred an additional consultation cost. All primary care consultations were assumed to be undertaken by a general practitioner (GP); a sensitivity analysis with 50% undertaken by a first contact physiotherapist practitioner (FCP) was conducted.

For patients undergoing primary care anti-CCP testing, 50% were assumed to receive concurrent routine C-reactive protein (CRP) and rheumatoid factor (RF) testing. For patients not undergoing primary care anti-CCP testing but referred to rheumatology services (e.g. following persistent symptoms), 50% were similarly assumed to have already had CRP and RF tests within primary care. For any referred patients in whom CRP and RF testing had not already been conducted, these were assumed to be undertaken within secondary care. Additional investigations (MRI, ultrasound and MRI) may also be undertaken at secondary care and were included in the model based on advice from the study advisory group clinicians [19].

For patients referred at a later primary care appointment, the total number of primary care appointments experienced was based on data from the Stack *et al.* dataset [14]. In the simulation, each patient’s primary care referral delay value was first sampled, with the number of primary care appointments subsequently estimated based on a simple regression model estimating the relationship between the number of appointments and the primary care referral delay (see Supplementary Data S1, available at *Rheumatology Advances in Practice* Online, for details).

#### Costs

The model includes direct healthcare costs relating to primary and secondary care tests and consultations. All costs were derived from the latest available National Cost Collection for the NHS and inflated to 2024 prices [20–22]. A sensitivity analysis was conducted using alternative (cheaper) costs for primary care tests, provided by the Leeds Teaching Hospitals NHS Trust (£5.55 for anti-CCP; £4.37 for CRP; £2.18 for RF). The anti-CCP prediction model was assumed to incur zero cost. Costs incurred in the model after 12 months (e.g. for patients experiencing long delays) were discounted at an annual rate of 3.5% per current guidelines [23].

#### Analysis

A probabilistic sensitivity analysis (PSA) was conducted using 5000 model runs, with each run evaluating outcomes for the hypothetical cohort of 10 000 patients but sampling different parameter input values based on assigned parameter distributions (Table 1). The model cohort and PSA sample size were selected to ensure stable model outputs and minimize Monte Carlo variability (simulation-related random fluctuations in model outputs) while maintaining computational feasibility, consistent with standard practice in economic modelling [24]. A range of sensitivity analyses were conducted to explore the impact of key parameters.

We report 95% uncertainty intervals (UIs) for each of the modelled outputs, calculated as the 2.5th–97.5th percentiles across the 5000 PSA simulations. These intervals reflect uncertainty arising from parameter uncertainty propagated through the model together with residual Monte Carlo variability, rather than patient sampling variability observed in clinical studies.

## Results

### Base-case analysis

Out of a modelled population of 10 000 patients with new onset non-specific MSK symptoms without synovitis, 190 patients are expected to develop IA (including RA) within 1 year of first presentation (based on a prevalence of 1.90%) [9]. Adopting increasing levels of anti-CCP testing means more of these patients are immediately referred—from 0% with no testing, to 52.7% (95% UI: 52.5–52.8%) when testing everyone on first presentation (Table 2). The proportion of imminent IA patients seen in rheumatology services within 12 weeks likewise increases, from 48.2% (48.2–48.3%) with no testing to 71.9% (71.8–72.1%) testing everyone (see Supplementary Table S1, available at *Rheumatology Advances in Practice* Online). However, due to the need for follow-up appointments in a proportion of those tested, the average number of primary care appointments for referred patients increases with indiscriminate anti-CCP testing—from 3.80 (3.79–3.81) with no testing to 4.05 (4.04–4.06) when testing everyone.

**Table 2:** Base-case results

Anti-CCP testing scenario	% receiving anti-CCP at 1st appointment	Secondary care referrals			Average number of primary care consultations for referred patients	Average per-patient cost (£) (discounted)	ICER* (vs. no anti-CCP testing)
		% of all patients referred immediately	% of imminent IA population referred immediately	% of all patients referred any time			
**No testing**	**0%**	**0%** (0, 0)	**0%** (0, 0)	**12.12%** (12.07, 12.17)	**3.80** (3.79, 3.81)	**£77.44** (77.12, 77.76)	–
**Anti-CCP testing**	**10%**	**0.25%** (0.25, 0.25)	**4.23%** (4.21, 4.25)	**11.97%** (11.92, 12.02)	**3.83** (3.82, 3.84)	**£80.89** (80.58, 81.19)	**£4287** (420, 8913)
	**20%**	**0.66%** (0.66, 0.66)	**11.82%** (11.78, 11.87)	**12.33%** (12.29, 12.37)	**3.83** (3.82, 3.84)	**£87.01** (86.73, 87.29)	**£4261** (1949, 7433)
	**30%**	**0.8%** (0.8, 0.81)	**17.45%** (17.39, 17.52)	**12.39%** (12.34, 12.43)	**3.87** (3.86, 3.88)	**£91.73** (91.46, 92.01)	**£4311** (2075, 7241)
	**40%**	**0.97%** (0.97, 0.97)	**20.26%** (20.19, 20.33)	**12.87%** (12.82, 12.91)	**3.90** (3.89, 3.91)	**£98.98** (98.68, 99.29)	**£5598** (3673, 8296)
	**50%**	**1.33%** (1.33, 1.33)	**28.9%** (28.81, 28.99)	**12.68%** (12.63, 12.74)	**3.92** (3.91, 3.93)	**£102.07** (101.72, 102.41)	**£4486** (2972, 6585)
	**75%**	**1.88%** (1.88, 1.89)	**36.46%** (36.35, 36.57)	**13.58%** (13.54, 13.62)	**4.00** (3.99, 4.01)	**£118.67** (118.35, 118.98)	**£5952** (4039, 8515)
	**100%**	**2.57%** (2.56, 2.57)	**52.65%** (52.49, 52.81)	**13.38%** (13.34, 13.43)	**4.05** (4.04, 4.06)	**£127.83** (127.49, 128.17)	**£5038** (3345, 7303)
**Targeted testing (with anti-CCP prediction model)**	**18.59 %** (18.57, 18.62)	**1.11%** (1.1, 1.11)	**23.81%** (23.72, 23.89)	**12.63%** (12.58, 12.67)	**3.75** (3.74, 3.76)	**£87.54** (87.25, 87.84)	**£2234** (1231, 3557)

* All parentheses show corresponding 95% uncertainty intervals, calculated as the 2.5th–97.5th percentiles across the 5000 modelled PSA simulations. IA, inflammatory arthritis; ICER, Incremental cost-effectiveness ratio; PSA, probabilistic sensitivity analysis; ‘Immediately referred’, patient is referred at their first primary care appointment. The ICER represents the average additional cost per additional immediate referral of a patient with imminent IA (IA or RA within 1 year), versus the ‘no testing’ scenario.

Targeted anti-CCP testing using the anti-CCP prediction model leads to 23.8% (95% UI: 23.7–23.9%) of imminent-IA patients being immediately referred, with 57.7% (55.7–55.8%%) seen within 12 weeks—similar outputs as achieved with indiscriminately testing 40% of patients with anti-CCP, but requiring less of the total population (18.6%) to be tested. The average number needed to test (NNT) to identify and refer one patient in the imminent IA population is more than halved when adopting targeted testing (NNT = 41) compared with indiscriminate testing of 40% (NNT = 88). The prediction model is also able to marginally reduce the average number of primary care appointments for referred patients to 3.75 (95% UI: 3.74–3.76), by focusing testing in the at-risk population (who would likely experience further primary care appointments if not immediately referred).

All testing scenarios increase healthcare costs, primarily due to the direct cost of anti-CCP testing, as well as slightly increased secondary care referral costs (see Fig. 3). With no testing, the average cost per-patient is £77.44 (95% UI: £77.12 to £77.76), compared with £127.83 (£127.49 to £128.17) when testing all patients or £87.54 (£87.25 to £87.84) with targeted testing. Over the modelled population of 10 000 patients, this equates to an additional average cost (versus no testing) of £503 900 if testing all patients or £101 000 with targeted testing. The additional cost per additional immediate referral in the imminent IA population (versus no testing) is lower when adopting targeted testing with an ICER of £2234 (£1231 to £3557), compared with universal anti-CCP testing with an ICER of £5038 (£3345 to £7303). Note that wider uncertainty is observed around the mean ICER values compared with the other model outputs (particularly for scenarios associated with small incremental differences) due to the mathematical properties of ICERs as ratios, which means that small variation in incremental referrals can produce large swings in the cost-per-referral ICER estimates.

**Figure 3:**
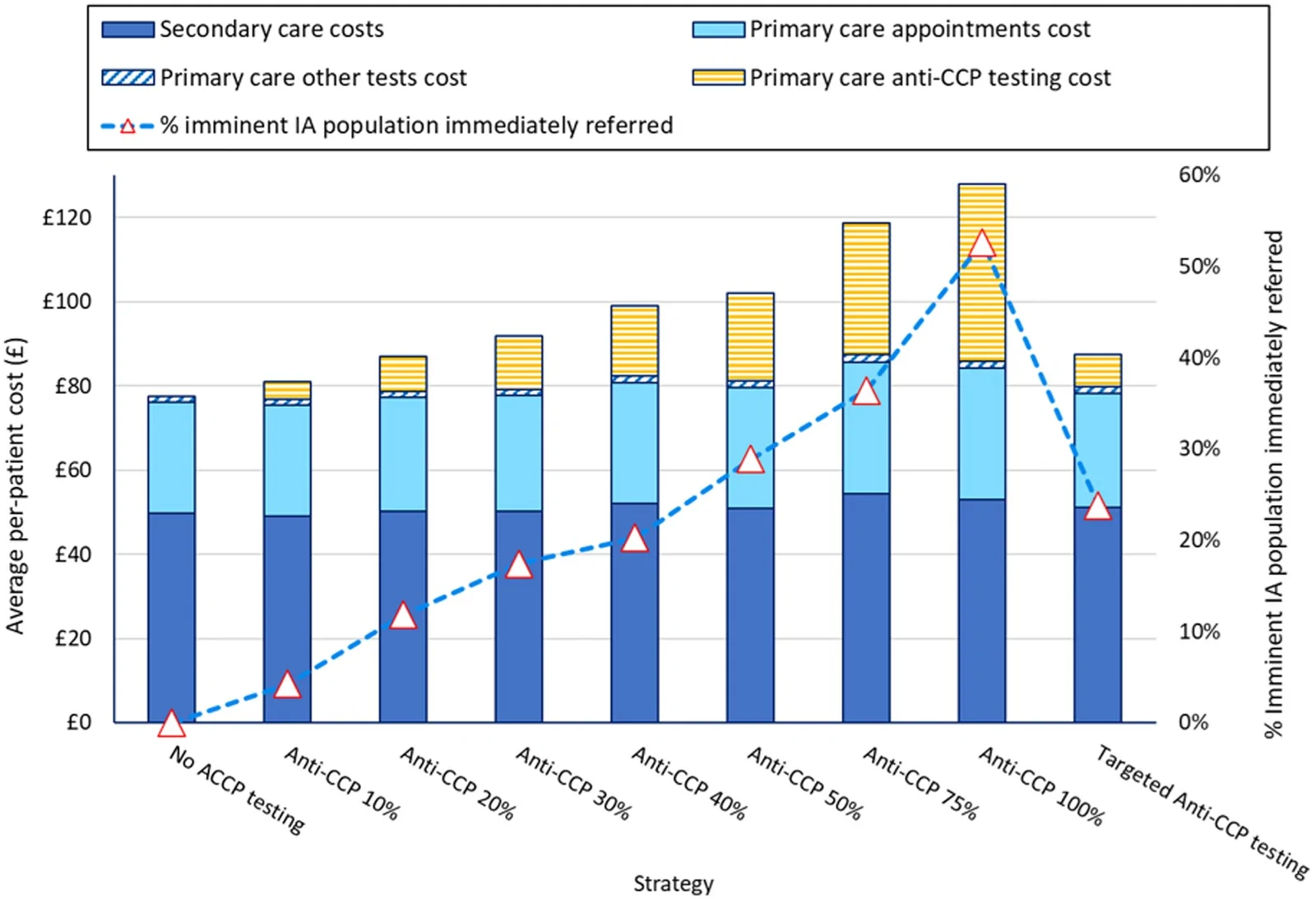
Base-case discounted average costs breakdown and percentage of imminent IA population immediately referred. The bars indicate the average per-patient costs (£) across alternative primary care testing scenarios. The bars are divided into key cost categories: primary care anti-CCP testing, primary care other tests, primary care appointments and secondary care costs (including secondary care referral appointments and tests). The overlayed line graph indicates, for each testing scenario, the percentage of patients with imminent IA (IA within 1 year) who are immediately referred under that scenario

### Sensitivity analyses

Most of the sensitivity analyses explored resulted in improved (lower) ICERs for the targeted testing strategy (Supplementary Tables S3–S6, available at *Rheumatology Advances in Practice* Online). The two exceptions were: (i) adopting a lower threshold for the anti-CCP prediction model (associated with higher sensitivity but lower specificity)—this increases the number of patients with imminent IA referred immediately (from 23.8% to 35.0%), but no longer maintains a reduction in the number of primary care appointments, resulting in higher costs and a slightly increased ICER of £2630 (still below all ICERs achieved with indiscriminate anti-CCP testing scenarios) (Supplementary Table S2, available at *Rheumatology Advances in Practice* Online) and (ii) doubling the proportion of patients assumed to return for a delayed referral, which increased the incremental costs associated with all testing scenarios, resulting in increased ICERs of £3476 (targeted testing) and £5217 (testing all) (Supplementary Table S7, available at *Rheumatology Advances in Practice* Online).

## Discussion

We used DES computer simulation methodology to explore the potential cost-effectiveness of targeted anti-CCP testing in the UK, using our previously developed anti-CCP prediction model. This methodology is well-suited for modelling patient pathways in healthcare, as it allows for individual variability in symptom presentation and referral delays.

Based on our findings, universal anti-CCP testing upon patients’ first primary care presentation is the best strategy to maximize early referrals in patients at risk of RA and minimize the risk of delayed diagnosis for patients whose high-risk status is missed by targeted testing. However, this is also the most expensive and resource-intensive strategy, which incurs a high volume of unnecessary tests and may not be feasible or appropriate in the NHS. Based on our exploratory analysis, targeted testing using the anti-CCP prediction model is more efficient, enabling early referral of around one-fifth of patients with imminent IA for an additional cost of £2234 per additional referral (versus no testing). Previous economic evaluations in this field have focused on early identification strategies in patients with pre-existing IA or undifferentiated IA [25–28]. To our knowledge, this is the first evaluation to focus specifically on patients with non-specific MSK symptoms without synovitis, for whom there is currently no clear strategy for early identification.

The utility of the targeted testing strategy depends on the clinical accuracy of the anti-CCP test and prediction model. In terms of sensitivity, both the prediction model (38.42%) and anti-CCP test itself (51.82%) may be considered low; nevertheless, against the standard-care comparator arm of *no* testing and *no* immediate referrals, a meaningful proportion of patients still can benefit under these testing scenarios in terms of reduced time to specialist rheumatology assessment. Our sensitivity analyses indicate that increasing the sensitivity of the prediction model can help to appropriately refer more patients, but this is at the expense of reduced specificity and results in higher costs and more primary care appointments compared with the base case prediction model scenario. This highlights the need to balance maximizing early intervention against available testing and appointment capacity when selecting referral thresholds.

A key limitation of our analysis is that we focused on short-term outcomes only. If increasing the number of at-risk patients seen promptly in secondary care can enable early intervention, then RA onset could potentially be delayed or prevented, thus preventing joint damage and disability, improving patients’ quality of life and reducing long-term treatment costs [3–5]. Two recent analyses from the Netherlands support the case for further research into early diagnosis and prevention strategies [6, 7]. The first, a cohort study of patients with confirmed RA, showed that patients with RA starting treatment early (<12 weeks from symptom onset) had markedly lower medication costs compared with those starting treatment later [6]. A second cost-utility analysis from the TREAT EARLIER randomized placebo-controlled trial (*n* = 236), found that treating patients with clinically suspect arthralgia who are at risk of developing RA with a 1-year course of methotrexate may marginally improve health outcomes and save healthcare and societal costs (although the cost savings were not statistically significant) [7]. In the context of primary care anti-CCP testing, further analysis is required to examine the knock-on impacts of early referrals in terms of costs and health-related quality of life, to provide a more definitive assessment of cost-effectiveness and identify an optimal testing strategy.

Our study found that the additional costs associated with testing could be reduced if patients can be managed by FCPs rather than GPs within primary care. This supports the findings of a recent UK analysis which found that FCPs offer a safe, clinically effective and cost-beneficial alternative to GP-led consultations for patients with MSK symptoms [29]. Given the current workload burden faced by GPs, greater utilization of FCP services for patients with MSK symptoms would appear to be a viable option to help alleviate GP pressures and may benefit patients.

Our analysis assumed that all patients with a positive anti-CCP result would be referred to rheumatology services. Recent analyses have also considered applying risk stratification models in this setting, as a means of focusing referrals and monitoring activities on those patients at highest risk of RA based on anti-CCP status combined with additional factors (such as RF, ESR, site of pain and smoking history) [9, 10]. Further analysis could consider utilizing such risk stratification models following anti-CCP testing, as a means of further minimizing unnecessary referrals and/or identifying appropriate risk-stratified monitoring strategies for those referred.

As with all economic decision models, this analysis is subject to limitations of the model—in particular the aforementioned focus on short-term impacts, as well as the dependency of the model on the quality of data and assumptions underpinning it. Data were lacking for key model parameters and assumptions had to be made, including regarding the proportion of patients expected to return for a delayed referral, the proportion of patients undergoing a follow-up appointment following an anti-CCP test and the time required for clinical review of test results. The key delay variables applied in the model were also based on data from patients who had confirmed IA, which may not be representative for patients who are at-risk but do not have IA. The modelled testing scenarios are also focused on patients who develop IA who are anti-CCP positive—some patients who test negative for anti-CCP may nevertheless go on to develop RA, and implementation of anti-CCP based strategies should consider safety-netting procedures for those patients testing negative, especially if they have new or persistent symptoms.

Our analysis focused on the UK-specific data, and the generalizability of the results should therefore be considered. Whilst parameters related to disease prevalence and test diagnostic performance may be expected to be broadly transferrable, those reflecting healthcare processes—particularly referral delays and access to rheumatology services—are likely to vary substantially across countries. The findings are therefore likely to be most comparable to other high-income healthcare systems that operate a primary care gatekeeper model, where access to specialist care is mediated through general practice and delays to referral and specialist assessment are common (e.g. the Netherlands, Scandinavian countries, Canada, Australia and New Zealand). While the absolute magnitude of benefit associated with different testing scenarios may differ across countries, the relative comparisons between testing scenarios and the insights regarding the potential value of earlier testing in primary care are likely to remain informative across similar healthcare systems.

Based on this early analysis, there is potential for the anti-CCP prediction model to provide a cost-effective means of identifying patients at risk of RA. These findings could inform NHS commissioning decisions regarding resource allocation for early RA identification strategies, including whether targeted anti-CCP testing should be integrated into MSK triage pathways. Implementation of any such strategy would depend on feasibility and service capacity considerations, including clinician acceptance and uptake of the prediction model and anti-CCP testing; workforce capacity and the potential role of First Contact Practitioners (FCPs) in delivering testing and triage; integration with electronic health record systems; and the capacity of laboratory and rheumatology services to accommodate increased testing and referral volumes.

## Supplementary Material

rkag111_Supplementary_Data

## Data Availability

The early economic model used in this study was developed using Simul8 software. The simulation code and associated documentation have been made publicly available in a GitHub repository to facilitate transparency and reproducibility. The repository can be accessed at: https://github.com/afc-smith/Target_RA_Model. The simulation model is available at: https://github.com/afc-smith/Target_RA_Model/releases/tag/v1.0.0. Data are available for the anti-CCP prediction model upon reasonable request by contacting the Leeds Co-ordinated Programme to Prevent Arthritis chief investigator (Paul Emery, e-mail: p.emery@leeds.ac.uk). Requests for access to the patient-level data from the Stack *et al.* 2019 study can be made to the study co-author (Rebecca Stack, email: r.stack@worc.ac.uk).
