# Supplementary material for: Targeted anti-CCP testing to identify people at risk of rheumatoid arthritis in primary care: an early economic evaluation
Source: Rheumatol Adv Pract. 2026 Sep 12;10(4):rkag111. doi: 10.1093/rap/rkag111 (PMC13626717; doi:10.1093/rap/rkag111)
Supplement: Supplementary file 1 [file rkag111_supplementary_data.docx]

**Supplementary Materials**

List of Supplementary materials:

- **Supplementary Figure S1.** Histogram and theoretical densities for patients’ primary care delay (for those referred after 1 appointment).
- **Supplementary Figure S2.** Histogram and theoretical densities for patients’ primary care delay (for those referred after 2 or more appointments).
- **Supplementary Figure S3.** Histogram and theoretical densities for patients’ secondary care (hospital) delay (for those referred).
- **Supplementary Data S1**. Further information on the GLM model
- **Supplementary Table S1.** Base-case analysis additional outcomes (average time to secondary care, and % of patients seen within 12 weeks of first primary care presentation)
- **Supplementary Table S2.** Alternative cut-off threshold for the anti-CCP prediction model (base-case = 11; sensitivity analysis = 8).
- **Supplementary Table S3.** Results for sensitivity analysis: Weibull parameterisation
- **Supplementary Table S4.** Results for sensitivity analysis: Alternative (cheaper) costs for primary care tests
- **Supplementary Table S5.** Results for sensitivity analysis: 50% of primary care appointments conducted by first contact physician (FCP) Physiotherapist
- **Supplementary Table S6.** Results for sensitivity analysis: halving rate of persistent symptoms
- **Supplementary Table S7.** Results for sensitivity analysis: doubling rate of persistent symptoms

Supplementary Figure S1. Histogram and theoretical densities for patients’ primary care delay (for those referred after 1 appointment).

Supplementary Figure S2. Histogram and theoretical densities for patients’ primary care delay (for those referred after 2 or more appointments).

Supplementary Figure S3. Histogram and theoretical densities for patients’ secondary care (hospital) delay (for those referred).

**Supplementary Data S1 – Further information on the simple regression model**

For patients referred at a later primary care appointment, the total number of primary care appointments experienced was based on data from the Stack et al dataset (14). In the simulation, each patient’s primary care referral delay value was first sampled, with the number of primary care appointments subsequently estimated based on a simple regression model estimating the relationship between the number of appointments and the primary care referral delay. The regression model is described in the following formula:

*N. primary care appointments = 3.536291 + [0.025208 * primary care referral delay]*

The regression model was fitted using R’s glm() function under the Gaussian family with identity link, which is equivalent to a simple linear regression. We used the Generalized Linear Model (GLM) framework for consistency and to facilitate extraction of the variance–covariance matrix for probabilistic sampling within the model. The model is bivariate, with one predictor (referral delay) and one outcome (number of primary care appointments). The GLM assumes a gaussian distribution and data on the variance and co-variance of the regression parameters was incorporated into the estimation. Sample values were drawn from a multivariate normal distribution using the coefficient values shown for the GLM parameter estimates and the co-variance matrix.

R output for GLM parameter estimates:

| **Variable** | **Estimate** | **Standard Error** | **T value** | **P value** |
| --- | --- | --- | --- | --- |
| Intercept | 3.54 | 0.35 | 10.13 | p < 0.001 |
| Referral Delay (wks) | 0.03 | 0.003 | 8.78 | p < 0.001 |

R Output for GLM parameter co-variance:

| **Parameter** | **Intercept** | **Referral Delay (weeks)** |
| --- | --- | --- |
| Intercept | 0.122 | -0.00033 |
| Referral Delay (weeks) | -0.00033 | 0.0000083 |

Note: values rounded to three significant figures.

Supplementary Table S1. Base-case analysis additional outcomes (average time to secondary care, and % of patients seen within 12 weeks of first primary care presentation)

| **Anti-CCP testing strategy** | **% receiving anti-CCP at 1st appointment** | **% of all patients referred immediately** | **% of imminent IA population referred immediately** | **% of all patients referred any time** | **Average time to secondary care for referred (weeks)** | **% seen within 12 weeks** | **% of imminent IA population seen within 12 weeks** |
| --- | --- | --- | --- | --- | --- | --- | --- |
| **No testing** | **0%** | **0%**  (0 , 0) | **0%**  (0, 0) | **12.12%**  (12.07, 12.17) | **13.67**  (13.67, 13.68) | **46.56%**  (46.54, 46.59) | **48.21%**  (48.15, 48.26) |
| **Anti-CCP testing** | **10%** | **0.25%**  (0.25, 0.25) | **4.23%**  (4.21, 4.25) | **11.97%**  (11.92, 12.02) | **13.49**  (13.49, 13.5) | **47.79%**  (47.77, 47.81) | **46.97%**  (46.91, 47.02) |
|  | **20%** | **0.66%**  (0.66, 0.66) | **11.82%**  (11.78, 11.87) | **12.33%**  (12.29, 12.37) | **13.66**  (13.66, 13.67) | **47.43%**  (47.41, 47.45) | **51.82%**  (51.77, 51.87) |
|  | **30%** | **0.8%**  (0.8, 0.81) | **17.45%**  (17.39, 17.52) | **12.39%**  (12.34, 12.43) | **13.38**  (13.38, 13.39) | **48.5%**  (48.47, 48.54) | **58.92%**  (58.84, 58.99) |
|  | **40%** | **0.97%**  (0.97, 0.97) | **20.26%**  (20.19, 20.33) | **12.87%**  (12.82, 12.91) | **13.33**  (13.32, 13.33) | **49.41%**  (49.38, 49.44) | **55.39%**  (55.33, 55.44) |
|  | **50%** | **1.33%**  (1.33, 1.33) | **28.9%**  (28.81, 28.99) | **12.68%**  (12.63, 12.74) | **12.97**  (12.97, 12.97) | **51.69%**  (51.66, 51.72) | **63.27%**  (63.19, 63.35) |
|  | **75%** | **1.88%**  (1.88, 1.89) | **36.46%**  (36.35, 36.57) | **13.58%**  (13.54, 13.62) | **12.86**  (12.86, 12.87) | **50.53%**  (50.51, 50.55) | **62.21%**  (62.13, 62.3) |
|  | **100%** | **2.57%**  (2.56, 2.57) | **52.65%**  (52.49, 52.81) | **13.38%**  (13.34, 13.43) | **12.22**  (12.21, 12.23) | **55.75%**  (55.7, 55.81) | **71.92%**  (71.8, 72.05) |
| **Targeted testing**  **(using anti-CCP prediction model)** | **18.59 %**  (18.57, 18.62) | **1.11%**  (1.1, 1.11) | **23.81%**  (23.72, 23.89) | **12.63%**  (12.58, 12.67) | **13.24**  (13.23, 13.24) | **49.68%**  (49.65, 49.71) | **57.71%**  (57.65, 57.77) |
| All parentheses show corresponding 95% uncertainty intervals, calculated as the 2.5^th­_^97.5^th^ percentiles across the 5,000 modelled PSA simulations. IA = inflammatory arthritis. ICER = Incremental cost-effectiveness ratio. PSA = probabilistic sensitivity analysis. ‘Immediately referred’ = patient is referred at their first primary care appointment. *The ICER represents the average additional cost per additional immediate referral of a patient with imminent IA (IA or RA within 1 year), versus the ‘no testing’ strategy. | | | | | | | |

Supplementary Table S2. Alternative cut-off threshold for the anti-CCP prediction model (base-case = 11; sensitivity analysis = 8).

| **Testing strategy** | **% receiving anti-CCP at 1st appointment** | **Secondary care referrals** | | | **Average number of primary care consultations for referred patients** | **Average per-patient cost (£) (discounted)** | **ICER***  **(vs. no testing)** |
| --- | --- | --- | --- | --- | --- | --- | --- |
|  |  | **% of all patients referred immediately** | **% of imminent IA population referred immediately** | **% of all patients referred any time** |  |  |  |
| **No testing** | **0%** | **0%**  (0 , 0) | **0%**  (0, 0) | **12.12%**  (12.07, 12.17) | **3.8**  (3.79, 3.81) | **£77.44**  (77.12, 77.76) | - |
| **Targeted testing**  **(anti-CCP prediction model threshold = 11)** | **18.56%** (18.54, 18.57) | **1.11%**  (1.1, 1.11) | **23.81%**  (23.72, 23.89) | **12.63%**  (12.58, 12.67) | **3.75**  (3.74, 3.76) | **£87.54**  (87.25, 87.84) | **£2,234**  (1231, 3557) |
| **Targeted testing**  **(anti-CCP prediction model threshold = 8)** | **39.97%** (39.95, 39.99) | **1.6%**  (1.59, 1.6) | **34.99%**  (34.88, 35.09) | **12.34%**  (12.3, 12.39) | **3.8**  (3.79, 3.81) | **£94.92**  (94.63, 95.22) | **£2,630**  (1584, 4097) |
| All parentheses show corresponding 95% uncertainty intervals, calculated as the 2.5^th­_^97.5^th^ percentiles across the 5,000 modelled PSA simulations. IA = inflammatory arthritis. ICER = Incremental cost-effectiveness ratio. PSA = probabilistic sensitivity analysis. ‘Immediately referred’ = patient is referred at their first primary care appointment. *The ICER represents the average additional cost per additional immediate referral of a patient with imminent IA (IA or RA within 1 year), versus the ‘no testing’ scenario. | | | | | | | |

Supplementary Table S3. Results for sensitivity analysis: Weibull parameterisation

| **Testing scenario** | **% receiving anti-CCP at 1st appointment** | **Secondary care referrals** | | | **Average number of primary care consultations for referred patients** | **Average per-patient cost (£) (discounted)** | **ICER***  **(vs. no testing)** |
| --- | --- | --- | --- | --- | --- | --- | --- |
|  |  | **% of all patients referred immediately** | **% of imminent IA population referred immediately** | **% of all patients referred any time** |  |  |  |
| **No testing** | **0%** | 0%  (0, 0) | 0%  (0, 0) | 12.27%  (12.23, 12.32) | 3.83  (3.81, 3.84) | £78.46  (78.16, 78.76) | - |
| **Anti-CCP testing** | **10%** | 0.21%  (0.21, 0.21) | 5.24%  (5.22, 5.26) | 12.28%  (12.23, 12.32) | 3.85  (3.84, 3.86) | £82.89  (82.61, 83.16) | £4,452  (1112, 9005) |
|  | **20%** | 0.52%  (0.52, 0.53) | 11.95%  (11.91, 12) | 12.24%  (12.19, 12.28) | 3.87  (3.86, 3.88) | £86.64  (86.36, 86.93) | £3,606  (1727, 5822) |
|  | **30%** | 0.8%  (0.8, 0.8) | 16.23%  (16.16, 16.3) | 12.81%  (12.77, 12.85) | 3.86  (3.85, 3.87) | £94.35  (94.08, 94.63) | £5,158  (3228, 7903) |
|  | **40%** | 1.18%  (1.18, 1.18) | 21.03%  (20.96, 21.1) | 12.96%  (12.92, 13) | 3.92  (3.91, 3.93) | £99.8  (99.51, 100.09) | £5,342  (3426, 7930) |
|  | **50%** | 1.39%  (1.39, 1.4) | 25.98%  (25.91, 26.06) | 13.07%  (13.03, 13.11) | 3.91  (3.9, 3.92) | £104.07  (103.79, 104.36) | £5,191  (3471, 7412) |
|  | **75%** | 2.14%  (2.14, 2.15) | 42.41%  (42.31, 42.52) | 13.2%  (13.15, 13.25) | 3.96  (3.95, 3.97) | £115.44  (115.1, 115.78) | £4,590  (3043, 6421) |
|  | **100%** | 2.82%  (2.82, 2.83) | 52.69%  (52.57, 52.81) | 13.47%  (13.43, 13.51) | 4.04  (4.03, 4.05) | £128.18  (127.84, 128.51) | £4,967  (3384, 6804) |
| **Targeted testing**  **(using anti-CCP prediction model)** | **18.59 %**  (18.57, 18.62) | 1.07%  (1.06, 1.07) | 23.26%  (23.17, 23.36) | 12.36%  (12.32, 12.4) | 3.79  (3.78, 3.8) | £86.42  (86.13, 86.71) | £1,802  (921, 3030) |
| All parentheses show corresponding 95% uncertainty intervals, calculated as the 2.5^th­_^97.5^th^ percentiles across the 5,000 modelled PSA simulations. IA = inflammatory arthritis. ICER = Incremental cost-effectiveness ratio. PSA = probabilistic sensitivity analysis. ‘Immediately referred’ = patient is referred at their first primary care appointment. *The ICER represents the average additional cost per additional immediate referral of a patient with imminent IA (IA or RA within 1 year), versus the ‘no testing’ scenario. | | | | | | | |

Supplementary Table S4. Results for sensitivity analysis: Alternative (cheaper) costs for primary care tests

| **Testing scenario** | **% receiving anti-CCP at 1st appointment** | **Secondary care referrals** | | | **Average number of primary care consultations for referred patients** | **Average per-patient cost (£) (discounted)** | **ICER***  **(vs. no testing)** |
| --- | --- | --- | --- | --- | --- | --- | --- |
|  |  | **% of all patients referred immediately** | **% of imminent IA population referred immediately** | **% of all patients referred any time** |  |  |  |
| **No testing** | **0%** | 0%  (0, 0) | 0%  (0, 0) | 12.12%  (12.07, 12.17) | 3.8  (3.79, 3.81) | £75.74  (75.42, 76.05) | - |
| **Anti-CCP testing** | **10%** | 0.25%  (0.25, 0.25) | 4.23%  (4.21, 4.25) | 11.97%  (11.92, 12.02) | 3.83  (3.82, 3.84) | £78.93  (78.63, 79.23) | £3,976  (225, 8474) |
|  | **20%** | 0.66%  (0.66, 0.66) | 11.82%  (11.78, 11.87) | 12.33%  (12.29, 12.37) | 3.83  (3.82, 3.84) | £84.74  (84.47, 85.02) | £4,009  (1721, 7200) |
|  | **30%** | 0.8%  (0.8, 0.81) | 17.45%  (17.39, 17.52) | 12.39%  (12.34, 12.43) | 3.87  (3.86, 3.88) | £89.17  (88.9, 89.43) | £4,050  (1940, 6930) |
|  | **40%** | 0.97%  (0.97, 0.97) | 20.26%  (20.19, 20.33) | 12.87%  (12.82, 12.91) | 3.9  (3.89, 3.91) | £96.08  (95.78, 96.38) | £5,286  (3365, 7917) |
|  | **50%** | 1.33%  (1.33, 1.33) | 28.9%  (28.81, 28.99) | 12.68%  (12.63, 12.74) | 3.92  (3.91, 3.93) | £98.91  (98.56, 99.25) | £4,221  (2743, 6222) |
|  | **75%** | 1.88%  (1.88, 1.89) | 36.46%  (36.35, 36.57) | 13.58%  (13.54, 13.62) | 4  (3.99, 4.01) | £114.7  (114.39, 115.01) | £5,625  (3755, 8062) |
|  | **100%** | 2.57%  (2.56, 2.57) | 52.65%  (52.49, 52.81) | 13.38%  (13.34, 13.43) | 4.05  (4.04, 4.06) | £123.18  (122.84, 123.51) | £4,744  (3115, 6836) |
| **Targeted testing**  **(using anti-CCP prediction model)** | **18.59 %**  (18.57, 18.62) | 1.11%  (1.1, 1.11) | 23.81%  (23.72, 23.89) | 12.63%  (12.58, 12.67) | 3.75  (3.74, 3.76) | £85.28  (84.99, 85.56) | £2,109  (1144, 3321) |
| All parentheses show corresponding 95% uncertainty intervals, calculated as the 2.5^th­_^97.5^th^ percentiles across the 5,000 modelled PSA simulations.. IA = inflammatory arthritis. ICER = Incremental cost-effectiveness ratio. PSA = probabilistic sensitivity analysis. ‘Immediately referred’ = patient is referred at their first primary care appointment. *The ICER represents the average additional cost per additional immediate referral of a patient with imminent IA (IA or RA within 1 year), versus the ‘no testing’ scenario. | | | | | | | |

Supplementary Table S5. Results for sensitivity analysis: 50% of primary care appointments conducted by first contact physician (FCP) Physiotherapist

| **Testing scenario** | **% receiving anti-CCP at 1st appointment** | **Secondary care referrals** | | | **Average number of primary care consultations for referred patients** | **Average per-patient cost (£) (discounted)** | **ICER***  **(vs. no testing)** |
| --- | --- | --- | --- | --- | --- | --- | --- |
|  |  | **% of all patients referred immediately** | **% of imminent IA population referred immediately** | **% of all patients referred any time** |  |  |  |
| **No testing** | **0%** | 0%  (0, 0) | 0%  (0, 0) | 12.12%  (12.07, 12.17) | 3.8  (3.79, 3.81) | £69.27  (68.99, 69.56) | - |
| **Anti-CCP testing** | **10%** | 0.25%  (0.25, 0.25) | 4.23%  (4.21, 4.25) | 11.97%  (11.92, 12.02) | 3.83  (3.82, 3.84) | £80.89  (80.58, 81.19) | £14,456  (9127, 23903) |
|  | **20%** | 0.66%  (0.66, 0.66) | 11.82%  (11.78, 11.87) | 12.33%  (12.29, 12.37) | 3.83  (3.82, 3.84) | £87.01  (86.73, 87.29) | £7,898  (5279, 11696) |
|  | **30%** | 0.8%  (0.8, 0.81) | 17.45%  (17.39, 17.52) | 12.39%  (12.34, 12.43) | 3.87  (3.86, 3.88) | £91.73  (91.46, 92.01) | £6,774  (4425, 10535) |
|  | **40%** | 0.97%  (0.97, 0.97) | 20.26%  (20.19, 20.33) | 12.87%  (12.82, 12.91) | 3.9  (3.89, 3.91) | £98.98  (98.68, 99.29) | £7,721  (5424, 11041) |
|  | **50%** | 1.33%  (1.33, 1.33) | 28.9%  (28.81, 28.99) | 12.68%  (12.63, 12.74) | 3.92  (3.91, 3.93) | £87.62  (87.31, 87.92) | £3,341  (2344, 4754) |
|  | **75%** | 1.88%  (1.88, 1.89) | 36.46%  (36.35, 36.57) | 13.58%  (13.54, 13.62) | 4  (3.99, 4.01) | £100.6  (100.34, 100.86) | £4,524  (3225, 6344) |
|  | **100%** | 2.57%  (2.56, 2.57) | 52.65%  (52.49, 52.81) | 13.38%  (13.34, 13.43) | 4.05  (4.04, 4.06) | £106.92  (106.65, 107.2) | £3,764  (2675, 5388) |
| **Targeted testing**  **(using anti-CCP prediction model)** | **18.59 %**  (18.57, 18.62) | 1.11%  (1.1, 1.11) | 23.81%  (23.72, 23.89) | 12.63%  (12.58, 12.67) | 3.75  (3.74, 3.76) | £77.05 (76.79, 77.31) | £1,720  (932, 2754) |
| All parentheses show corresponding 95% uncertainty intervals, calculated as the 2.5^th­_^97.5^th^ percentiles across the 5,000 modelled PSA simulations. IA = inflammatory arthritis. ICER = Incremental cost-effectiveness ratio. PSA = probabilistic sensitivity analysis. ‘Immediately referred’ = patient is referred at their first primary care appointment. *The ICER represents the average additional cost per additional immediate referral of a patient with imminent IA (IA or RA within 1 year), versus the ‘no testing’ scenario. | | | | | | | |

Supplementary Table S6. Results for sensitivity analysis: halving rate of persistent symptoms

| **Testing scenario** | **% receiving anti-CCP at 1st appointment** | **Secondary care referrals** | | | **Average number of primary care consultations for referred patients** | **Average per-patient cost (£) (discounted)** | **ICER***  **(vs. no testing)** |
| --- | --- | --- | --- | --- | --- | --- | --- |
|  |  | **% of all patients referred immediately** | **% of imminent IA population referred immediately** | **% of all patients referred any time** |  |  |  |
| **No testing** | **0%** | 0%  (0, 0) | 0%  (0, 0) | 7.05%  (7.03, 7.07) | 3.79  (3.78, 3.8) | £45.17  (45.04, 45.29) | - |
| **Anti-CCP testing** | **10%** | 0.29%  (0.29, 0.29) | 4.86%  (4.84, 4.88) | 7.25%  (7.23, 7.27) | 3.77  (3.76, 3.78) | £50.21  (50.06, 50.36) | £5,460  (2094, 9894) |
|  | **20%** | 0.59%  (0.59, 0.6) | 9.79%  (9.75, 9.82) | 7.63%  (7.61, 7.65) | 3.76  (3.75, 3.77) | £56.64  (56.48, 56.8) | £6,170  (3478, 9503) |
|  | **30%** | 0.79%  (0.79, 0.79) | 16.34%  (16.29, 16.4) | 7.58%  (7.56, 7.61) | 3.77  (3.76, 3.78) | £60.69  (60.53, 60.86) | £5,001  (3179, 7861) |
|  | **40%** | 0.95%  (0.95, 0.96) | 16.72%  (16.66, 16.79) | 7.67%  (7.64, 7.69) | 3.79  (3.79, 3.8) | £65.14  (64.96, 65.31) | £6,287  (3914, 9796) |
|  | **50%** | 1.3%  (1.29, 1.3) | 26.5%  (26.42, 26.58) | 7.75%  (7.73, 7.77) | 3.78  (3.77, 3.79) | £69.87  (69.71, 70.04) | £4,908  (3239, 7,169) |
|  | **75%** | 1.85%  (1.84, 1.86) | 36.55%  (36.43, 36.66) | 8.22%  (8.2, 8.25) | 3.74  (3.74, 3.75) | £82.85  (82.65, 83.05) | £5,428  (3603, 8132) |
|  | **100%** | 2.56%  (2.55, 2.57) | 52.82%  (52.69, 52.95) | 8.54%  (8.52, 8.57) | 3.76  (3.75, 3.77) | £95.55  (95.34, 95.77) | £5,022  (3434, 7002) |
| **Targeted testing**  **(using anti-CCP prediction model)** | **18.59 %**  (18.57, 18.62) | 1%  (1, 1) | 24.69%  (24.61, 24.78) | 7.62%  (7.6, 7.64) | 3.65  (3.64, 3.66) | £55.58  (55.45, 55.72) | £2,221  (1355, 3423) |
| All parentheses show corresponding 95% uncertainty intervals, calculated as the 2.5^th­_^97.5^th^ percentiles across the 5,000 modelled PSA simulations. IA = inflammatory arthritis. ICER = Incremental cost-effectiveness ratio. PSA = probabilistic sensitivity analysis. ‘Immediately referred’ = patient is referred at their first primary care appointment. *The ICER represents the average additional cost per additional immediate referral of a patient with imminent IA (IA or RA within 1 year), versus the ‘no testing’ scenario. | | | | | | | |

Supplementary Table S7. Results for sensitivity analysis: doubling rate of persistent symptoms

| **Testing scenario** | **% receiving anti-CCP at 1st appointment** | **Secondary care referrals** | | | **Average number of primary care consultations for referred patients** | **Average per-patient cost (£) (discounted)** | **ICER***  **(vs. no testing)** |
| --- | --- | --- | --- | --- | --- | --- | --- |
|  |  | **% of all patients referred immediately** | **% of imminent IA population referred immediately** | **% of all patients referred any time** |  |  |  |
| **No testing** | **0%** | 0%  (0, 0) | 0%  (0, 0) | 21.89%  (21.81, 21.97) | 3.8  (3.79, 3.81) | £139.83  (139.3, 140.37) | - |
| **Anti-CCP testing** | **10%** | 0.28%  (0.28, 0.28) | 6.6%  (6.57, 6.63) | 22.42%  (22.33, 22.5) | 3.86  (3.84, 3.87) | £147.98  (147.41, 148.54) | £6,495  (3321, 11244) |
|  | **20%** | 0.56%  (0.56, 0.56) | 11.68%  (11.63, 11.73) | 22.37%  (22.27, 22.46) | 3.9  (3.89, 3.91) | £152.02  (151.4, 152.64) | £5,493  (2386, 10342) |
|  | **30%** | 0.87%  (0.87, 0.87) | 19.19%  (19.14, 19.24) | 22.12%  (22.03, 22.21) | 3.94  (3.93, 3.95) | £154.8  (154.19, 155.41) | £4,106  (2031, 7012) |
|  | **40%** | 1.05%  (1.05, 1.06) | 25.03%  (24.96, 25.1) | 22.01%  (21.93, 22.1) | 3.98  (3.97, 3.99) | £158.65  (158.06, 159.23) | £3,957  (2297, 6085) |
|  | **50%** | 1.24%  (1.24, 1.24) | 28.38%  (28.29, 28.47) | 22.58%  (22.49, 22.67) | 4.05  (4.04, 4.06) | £166.97  (166.34, 167.59) | £5,034  (3171, 7583) |
|  | **75%** | 1.79%  (1.79, 1.8) | 36.95%  (36.84, 37.07) | 22.73%  (22.63, 22.82) | 4.17  (4.16, 4.18) | £179.28  (178.65, 179.91) | £5,621  (3520, 8591) |
|  | **100%** | 2.41%  (2.4, 2.42) | 52.95%  (52.8, 53.1) | 22.93%  (22.84, 23.03) | 4.29  (4.28, 4.3) | £192.51  (191.86, 193.16) | £5,238  (3506, 7561) |
| **Targeted testing**  **(using anti-CCP prediction model)** | **18.59 %**  (18.57, 18.62) | 1.08%  (1.08, 1.09) | 22.41%  (22.33, 22.5) | 22.3%  (22.21, 22.39) | 3.83  (3.82, 3.84) | £149.83  (149.23, 150.42) | £2,348  (943, 4555) |
| All parentheses show corresponding 95% uncertainty intervals, calculated as the 2.5^th­_^97.5^th^ percentiles across the 5,000 modelled PSA simulations. IA = inflammatory arthritis. ICER = Incremental cost-effectiveness ratio. PSA = probabilistic sensitivity analysis. ‘Immediately referred’ = patient is referred at their first primary care appointment. *The ICER represents the average additional cost per additional immediate referral of a patient with imminent IA (IA or RA within 1 year), versus the ‘no testing’ scenario. | | | | | | | |
